# RGB-Based Visual–Inertial Odometry via Knowledge Distillation from Self-Supervised Depth Estimation with Foundation Models

**DOI:** 10.3390/s25175366

**Published:** 2025-08-30

**Authors:** Jimin Song, Sang Jun Lee

**Affiliations:** Division of Electronic Engineering, Jeonbuk National University, 567 Baekje-daero, Deokjin-gu, Jeonju 54896, Republic of Korea; jimin_song@jbnu.ac.kr

**Keywords:** simultaneous localization and mapping, visual–inertial odometry, deep learning, foundation model, self-supervised depth estimation, knowledge distillation

## Abstract

Autonomous driving represents a transformative advancement with the potential to significantly impact daily mobility, including enabling independent vehicle operation for individuals with visual disabilities. The commercialization of autonomous driving requires guaranteed safety and accuracy, underscoring the need for robust localization and environmental perception algorithms. In cost-sensitive platforms such as delivery robots and electric vehicles, cameras are increasingly favored for their ability to provide rich visual information at low cost. Despite recent progress, existing visual–inertial odometry systems still suffer from degraded accuracy in challenging conditions, which limits their reliability in real-world autonomous navigation scenarios. Estimating 3D positional changes using only 2D image sequences remains a fundamental challenge primarily due to inherent scale ambiguity and the presence of dynamic scene elements. In this paper, we present a visual–inertial odometry framework incorporating a depth estimation model trained without ground-truth depth supervision. Our approach leverages a self-supervised learning pipeline enhanced with knowledge distillation via foundation models, including both self-distillation and geometry-aware distillation. The proposed method improves depth estimation performance and consequently enhances odometry estimation without modifying the network architecture or increasing the number of parameters. The effectiveness of the proposed method is demonstrated through comparative evaluations on both the public KITTI dataset and a custom campus driving dataset, showing performance improvements over existing approaches.

## 1. Introduction

Recent advances in deep learning, driven by improvements in both hardware and software, have enabled its widespread application across various industries. In the field of computer vision, significant progress has been made not only in well-established areas such as object detection [1] and semantic segmentation [2], but also in depth estimation [3]. Depth estimation is the task of inferring the real-world distance from the camera to the scene surface at each pixel. This technique has become key for applications in robotics, including the autonomous navigation of mobile robots and drones [4,5], as well as for providing auxiliary information in minimally invasive surgery [6]. Due to its broad applicability across various domains, depth estimation continues to receive increasing attention. Although depth estimation has seen significant progress, it still faces fundamental challenges. Monocular methods are limited by scale ambiguity, making absolute depth recovery ill-posed without additional cues. In contrast, stereo approaches must trade off between long-range accuracy and occlusion handling, which depends on the baseline length. In this paper, we propose a novel monocular depth estimation method based on a foundation model, which is aimed at improving the performance of simultaneous localization and mapping (SLAM).

As a data-driven approach, the performance of deep neural networks is largely determined by the quality and quantity of the training data. Constructing high-quality datasets typically requires ensuring domain diversity, filtering out noisy or erroneous samples, and generating accurate ground-truth annotations. In recent years, increasing the awareness of ethical and legal concerns—such as privacy and copyright issues—has made data collection more cautious. To address these challenges, recent research has increasingly focused on the use of foundation models. Foundation models refer to large-scale models pretrained using extensive computational resources and massive datasets. In this work, we propose a method that fine-tunes a depth foundation model pretrained on a large-scale dataset comprising synthetic data generated from carefully designed virtual environments, using a target dataset.

**Figure 1:**
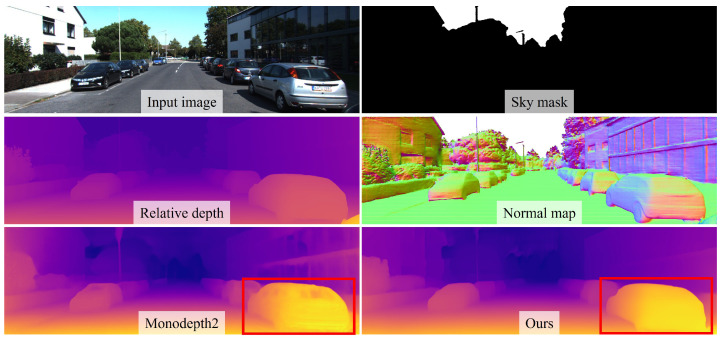
Geometric cues used for knowledge distillation and their effects. Sky mask, relative depth, and surface normal are derived using foundation model-based methods [7,8,9]. These geometric cues guide our knowledge distillation framework, which outperforms [10] particularly in handling transparent objects challenging for reprojection-based approaches, as highlighted in the red box.

A straightforward and widely used approach for training depth estimation models is the supervised learning pipeline. This method minimizes a loss function defined based on the error between the estimated depth map and the ground-truth depth map. It typically yields high performance, as the model is trained directly using explicit supervisory signals. However, generating accurate ground-truth depth data requires expensive light detection and ranging (LiDAR) sensor and precise calibration between sensors. Moreover, such ground-truth data are often domain-specific, leading to overfitting to the data acquisition environment and resulting in limited generalization performance. As an alternative, self-supervised learning approaches based on image reprojection have been proposed to mitigate these limitations. These methods estimate a depth map from an input image and leverage adjacent frames to reproject their content into the viewpoint of the input image, using the photometric error between the synthesized and original views as the supervision signal. Due to their complex and indirect supervision pipelines, self-supervised methods typically exhibit lower accuracy than supervised approaches. To address this limitation, we introduce a self-supervised framework that improves depth estimation by incorporating auxiliary depth cues via knowledge distillation, as shown in Figure 1, while preserving image reprojection-based supervision. Knowledge distillation refers to the transfer of learned representations from a teacher model to a student model.

SLAM is a technique that enables a robot to simultaneously construct a map of its environment and estimate its position within it in real time. The performance of SLAM algorithms is primarily influenced by the sensor modality. Recent advancements in SLAM research have given rise to two primary system categories: LiDAR-based approaches and camera-based approaches. LiDAR-based methods are known for their high accuracy, which is primarily due to their capability to directly capture detailed and reliable 3D points. However, they face several challenges, including high cost and reduced reliability in environments such as highways, which are characterized by repetitive or low-texture geometric features. Additionally, they require extra processing to mitigate issues caused by light reflection and material transparency. Camera-based SLAM systems, on the other hand, are generally more robust in such scenarios and offer a more cost-effective alternative. Nevertheless, due to the absence of direct 3D measurements, camera-based SLAM systems generally underperform compared to LiDAR-based approaches. In this work, we demonstrate that the proposed camera-based SLAM system with integrated depth estimation outperforms existing methods on a real-world outdoor dataset.

To summarize, the key contributions of this work are as follows: (1) We propose a knowledge distillation framework to enhance the capability of fine-detail recovery in self-supervised monocular depth estimation, addressing a common shortcoming observed in previous approaches. Our approach incorporates not only recently proposed self-distillation techniques but also introduces a novel geometry-aware distillation method leveraging a foundation depth model. (2) To ensure the practical deployment of the trained student depth model in visual–inertial odometry systems, we adopt a ViT-Small-based architecture. Compared to ViT-Large, our model significantly reduces the number of parameters with only marginal accuracy loss. Furthermore, it outperforms simple fine-tuning baselines in reconstructing fine details and estimating reasonable depth for transparent objects. (3) We extensively evaluate our approach on both public datasets and real-world outdoor driving sequences collected in house, demonstrating its effectiveness in both depth estimation and odometry performance compared to existing methods.

## 2. Related Work

Monodepth2 [10] serves as a commonly used baseline in recent self-supervised depth estimation research. Godard et al. introduced a method to ensure that only pixels satisfying the photometric consistency assumption in reprojection-based training are used for supervision. To address occlusion issues, they introduced a loss function that warps multiple source images into a single target image and computes the reprojection error, selecting only the minimum error across the sources. In addition, they proposed an automatic masking strategy to exclude pixels that violate the parallax assumption, such as those belonging to static background regions. In some cases, this strategy may also filter out moving objects whose motion is consistent with the camera, as these can otherwise degrade the quality of the supervision signal. These contributions enhanced the reliability of the underlying assumptions in the training pipeline and led to significant performance improvements. In contrast to previous methods that primarily suppress unreliable training signals, we introduce a knowledge distillation framework designed to provide more direct and semantically enriched supervision for self-supervised depth estimation.

Knowledge distillation has been extensively studied in deep learning across various tasks, including depth estimation. In the field of depth estimation, knowledge distillation has been applied both to reduce model complexity [11] and to transfer informative geometric cues [12] that contribute to improved depth prediction performance. In recent study, Poggi et al. [13] proposed a self-distillation framework where the student shares the same structure as the teacher and can even outperform it. Their method incorporates pixel-level uncertainty into the loss function via a negative log-likelihood formulation, allowing the student model to account for uncertainty during training. Song et al. [14] designed a modified model architecture and loss function tailored for the effective knowledge distillation of a foundation depth model. Their method demonstrated state-of-the-art performance on the KITTI online benchmark, providing empirical evidence of the strong generalization capability of foundation models when used as sources of transferable knowledge. Foundation models often require networks with a large number of parameters to fully leverage their representational capacity. However, practical applications such as SLAM benefit from models with significantly fewer parameters due to computational and resource constraints. To bridge this gap, we propose a knowledge distillation framework in which a lightweight student model is guided by both segmentation and depth foundation models through geometry-aware supervision.

Foundation models were initially developed for natural language processing tasks and have subsequently exhibited substantial impact in computer vision. Oquab et al. introduced DINOv2 [15], which is a robust vision transformer capable of extracting generalizable visual features from unseen images, enabling its use across various downstream tasks such as classification, segmentation, and depth estimation. Liu et al. proposed GroundingDINO [7], a vision-language model designed for object detection tasks involving unseen classes, which leverages diverse forms of text prompts to achieve strong performance. Kirillov et al. introduced the Segment Anything Model (SAM) [8], which is a prompt-driven segmentation framework capable of processing various input types—such as points, bounding boxes, masks, and text—alongside image data. Following recent work [16], we employ GroundingDINO and SAM to explicitly identify sky regions, which are unsuitable for quantitative evaluation and may adversely affect depth model training. Ranftl et al. proposed the Dense Prediction Transformer (DPT) [17], which is trained on a large meta-dataset constructed by aggregating multiple existing depth datasets. Yang et al. introduced Depth Anything v2 [9], a model composed of a DINOv2-based encoder and a DPT-based decoder, which is trained using both labeled synthetic images and unlabeled real-world images to enhance generalization. The foundation depth model can reliably estimate relative depth even for unseen images; however, fine tuning is required to achieve accurate absolute depth estimation. To address this limitation, we do not directly incorporate the absolute outputs of the foundation model into the knowledge distillation process. Instead, a surface normal map is derived from relative depth prediction and incorporated as auxiliary geometric supervision in the training framework.

SLAM has increasingly integrated Inertial Measurement Unit (IMU) sensors, which provide measurements of linear acceleration and angular velocity. While IMUs provide valuable measurements, they are prone to cumulative drift due to inherent sensor noise characteristics. Therefore, integrating complementary sensor modalities is often necessary to improve the robustness of state estimation. Representative examples include LiDAR–Inertial Odometry (LIO), which integrates LiDAR and IMU data, and Visual–Inertial Odometry (VIO), which combines camera and IMU measurements. Bai et al. proposed a Faster-LIO [18], introducing efficient data structures for handling point cloud representations. Their method offers computational efficiency while maintaining reliable and accurate state estimation. In this study, it is utilized to generate ground-truth odometry for our custom dataset. This enables a quantitative evaluation of odometry accuracy in VIO. Qin et al. introduced VINS-Mono [19], which is a widely adopted baseline VIO framework that fuses monocular images with IMU data. Building upon the original VINS-Mono framework, Shan et al. introduced VINS-RGBD [20], which incorporates depth data from an RGB-D camera. Leveraging prior frameworks, we propose an RGB-based VIO pipeline guided by foundation models, which was designed to enhance the robustness of vision-based autonomous navigation systems.

## 3. Method

**Figure 2:**
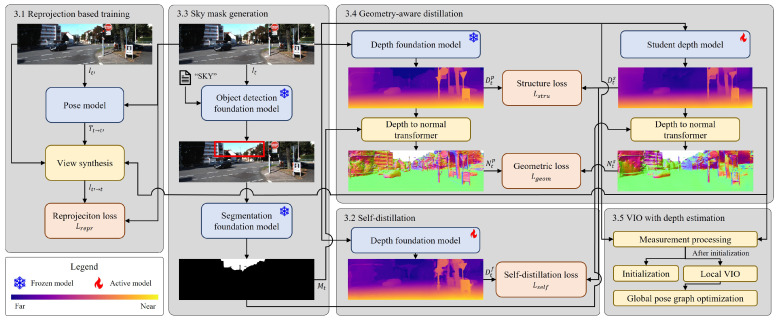
Overview of the proposed visual–inertial odometry pipeline. Yellow boxes represent non-learnable geometric modules, blue boxes indicate deep neural networks, and red boxes denote loss functions used for training the student network. In particular, geometry-aware distillation serves as a key module for learning the geometric consistency of the pretrained foundation model.

A detailed explanation of each component in the proposed VIO pipeline is presented in this section, as shown in Figure 2. Section 3.1 and Section 3.2 describe the image reprojection-based supervision strategy and the self-distillation scheme using a transformer-based foundation depth model. Section 3.3 describes the process of generating sky segmentation masks from foundation models for use in geometry-aware distillation. Section 3.4 presents a geometry-aware distillation strategy that transfers boundary and geometric cues from a pretrained foundation model to enhance student prediction accuracy. Finally, Section 3.5 presents the integration of the student depth model into a VIO pipeline.

### 3.1. Image Reprojection Based Training

Our proposed depth estimation training pipeline incorporates an image reprojection-based self-supervised learning strategy, following prior successful work in the field [10]. Given a target image $I_{t}$, a corresponding depth map $D_{t}^{s}$ is predicted by the student depth model. The relative camera transformation from the target view $I_{t}$ to a reference view $I_{t^{\prime }}$ is denoted as $T_{t\to t^{\prime }}$ to facilitate reprojection. In stereo settings, this transformation is derived from known extrinsic calibration parameters. In monocular settings, it is estimated by a ResNet [21]-based pose network that takes the image pair $\left(I_{t},I_{t^{\prime }}\right)$ as input. Given the camera intrinsic matrix *K*, the reprojected view $I_{t^{\prime }\to t}$ is synthesized from the reference image $I_{t^{\prime }}$.

(1) $$I_{t^{\prime }\to t}=I_{t^{\prime }}\langle \mathrm{proj}\left(D_{t}^{s},T_{t\to t^{\prime }},K\right)\rangle .$$

Here, proj(·) denotes the projection operation into the target frame, and 〈·〉 indicates bilinear sampling. A photometric loss $L_{p}$ is employed, combining the Structural Similarity Index Measure (SSIM) [22] and the L1-norm with a weighting factor α:

(2) $$L_{p}=\min_{t^{\prime }}\;pe\left(I_{t},I_{t^{\prime }\to t}\right),pe\left(I_{t},I_{t^{\prime }\to t}\right)=\frac{\alpha }{2}\left(1-\mathrm{SSIM}\left(I_{t},I_{t^{\prime }\to t}\right)\right)+\left(1-\alpha \right){∥I_{t}-I_{t^{\prime }\to t}∥}_{1}.$$

To account for occlusions, the minimum photometric error is computed across multiple reference frames, ensuring supervision is derived from the most photometrically consistent view. Since this approach assumes a static scene with a moving camera, a binary mask μ is introduced to restrict the loss computation to valid regions. It is computed as follows:

(3) $$\mu =\left[\min_{t^{\prime }}\;pe\left(I_{t},I_{t^{\prime }\to t}\right)<\min_{t^{\prime }}\;pe\left(I_{t},I_{t^{\prime }}\right)\right],$$

where [·] denotes the Iverson bracket. And we adopted an auxiliary loss term known as the edge-aware smoothness loss $L_{s}$, which encourages depth smoothness in textureless regions while preserving object boundaries:

(4) $$L_{s}=|\partial_{x}D_{t}^{s}|e^{-|\partial_{x}I_{t}|}+\left|\partial_{y}D_{t}^{s}\right|e^{-|\partial_{y}I_{t}|}.$$

Here, $\partial_{x}$ and $\partial_{y}$ denote the partial derivatives with respect to the *x*-axis and *y*-axis, respectively. The final reprojection loss is defined as a weighted combination of the masked photometric loss and the smoothness loss:

(5) $$L_{repr}=\mu L_{p}+\beta L_{s}.$$

In all experiments, the hyperparameter β is empirically set to 0.001. The reprojection-based approach is supported by established theoretical principles and demonstrates reliable performance in coarse depth estimation. However, it tends to struggle with fine-grained details such as object boundaries and transparent surfaces. This is because masking strategies such as Equation (3) and smoothness losses like Equation (4) aim to exclude cases that violate the underlying assumptions of the reprojection principles, which can lead to a lack of learning cues in those regions. To address these limitations, two knowledge distillation techniques are additionally employed, as detailed in the following sections.

### 3.2. Self-Distillation of Dense Prediction Transformer

Recent approaches [13,14] have explored self-distillation techniques in which a model fine-tuned on the target dataset is used to generate pseudo-ground truth depth map $D_{t}^{f}$. The pseudo-label is then used as a supervision signal to train the student model by minimizing a depth loss function. Although the generated pseudo-depth may be imperfect, the availability of dense supervision provides explicit guidance during training, often resulting in improved performance. Prior studies have shown that even when the teacher and student models share identical architectures, the student model can achieve superior performance through self-distillation. In contrast, our work aims to bridge the performance gap between a high-capacity teacher model and a lightweight student model with significantly fewer parameters. The depth models adopt the architecture of a depth foundation model based on DPT [17], utilizing a ViT-based encoder [23] to extract rich visual representations. The decoder consists of a reassemble block and a fusion block, which is followed by a prediction head that reconstructs the depth map in the image space. To better leverage dense depth cues, the student model is augmented with parameter-shared multi-prediction heads. Deep supervision is applied to all intermediate predictions to encourage the extraction of semantically meaningful features. For the self-distillation loss, we adopt the scale-invariant error, a widely used metric in supervised depth estimation [3], which compares the predicted depth $D_{t}^{s}$ with the pseudo-ground truth $D_{t}^{f}$ in logarithmic space:

(6) $$L_{self}=\frac{1}{n}\sum_{i=1}^{n}{\epsilon_{i}}^{2}-\frac{\lambda }{n^{2}}{\left(\sum_{i=1}^{n}\epsilon_{i}\right)}^{2},\epsilon_{i}=\log d_{i}^{s}-\log d_{i}^{f}.$$

Here, *n* denotes the number of valid pixels, while $d_{i}^{s}$ and $d_{i}^{f}$ represent the predicted and pseudo-depth values at pixel *i*.

### 3.3. Sky Mask Generation via Foundation Model

In depth estimation, the sky region is inherently unsuitable for accurate prediction due to its near-infinite depth, which fundamentally differs from other regions that exhibit observable geometric structure. Recent approaches, such as Depth Anything v2 [9], address this issue by training models to predict values that are inversely proportional to depth, thereby encouraging near-zero outputs for sky pixels. However, when applying similar strategies to a relatively limited real-world custom dataset, we observed a substantial degradation in both quantitative accuracy and generalization performance. Therefore, a sky segmentation mask $M_{t}$ is generated for input image $I_{t}$ to explicitly exclude these regions from the distillation process. The mask $M_{t}$ is generated in two stages, beginning with the input of the RGB image $I_{t}$ and the textual prompt “sky” into GroundingDINO [7], which is a state-of-the-art open-vocabulary object detection model recognized for its strong performance in zero-shot scenarios. This model produces bounding boxes that roughly localize sky regions. These bounding boxes then serve as strong prompts for SAM [8], which takes the RGB image $I_{t}$ and outputs a high-quality segmentation mask corresponding to the detected sky area.

### 3.4. Geometry-Aware Knowledge Distillation

Depth foundation models pretrained on large-scale datasets are capable of producing sharp and geometrically consistent relative depth maps $D_{t}^{p}$, even for previously unseen images. Leveraging this capability, we propose two geometry-aware distillation losses designed to transfer the boundary-aware and surface-consistent knowledge from the pretrained model to the student model. We introduce a structure consistency loss $L_{tru}$, which promotes edge preservation by evaluating local structural patterns instead of penalizing absolute depth differences. Inspired by the structural similarity term in the SSIM metric, the loss measures local geometric coherence between the foundation model prediction $D_{t}^{p}$ and the student output $D_{t}^{s}$.

(7) $$\begin{array}{c} L_{stru}=\frac{1}{n}\sum_{i=1}^{n}\left(1-\frac{\sigma_{i}^{ps}+k}{\sigma_{i}^{p}\sigma_{i}^{s}+k}\right), \end{array}$$

where $\sigma_{i}^{ps}$ denotes the local covariance between $D_{t}^{p}$ and $D_{t}^{s}$ at pixel *i*, while $\sigma_{i}^{p}$ and $\sigma_{i}^{s}$ represent the local standard deviations of $D_{t}^{p}$ and $D_{t}^{s}$, respectively. These statistics are computed over a 3×3 window centered at each pixel. The constant *k*, which is empirically set to 30 in accordance with the SSIM formulation, stabilizes the computation by preventing division by near-zero values.

To enhance the model’s understanding of object shapes through geometric context, we incorporate surface normal supervision, which provides richer structural information than depth values alone. To generate surface normal supervision, we employ Depth-to-Normal Transformer (D2NT) [24], which is a recently proposed method that achieves both high accuracy and computational efficiency. As normal estimation directly impacts the training speed of our pipeline, fast and reliable normal computation is a critical requirement. Both $D_{t}^{p}$ and $D_{t}^{s}$, along with the corresponding sky mask $M_{t}$, are passed through D2NT to produce surface normal maps $N_{t}^{p}$ and $N_{t}^{s}$, respectively. We then define a geometric consistency loss $L_{geom}$ based on cosine similarity between the predicted and reference normal vectors:

(8) $$\begin{array}{c} L_{geom}=\frac{1}{m}\sum_{i=1}^{m}\left(1-n_{i}^{p}\cdot n_{i}^{s}\right), \end{array}$$

where *m* denotes the number of pixels outside the masked region defined by $M_{t}$, and $n_{i}^{p}$ and $n_{i}^{s}$ represent the normal vectors at pixel *i* in $N_{t}^{p}$ and $N_{t}^{s}$, respectively.

By incorporating both boundary-sensitive and surface-aware supervision, the proposed method facilitates enhanced structural understanding within the student network, thereby promoting more stable and accurate depth estimation. Consequently, the total loss used for training the student depth model is formulated as a weighted combination of the following components:

(9) $$\begin{array}{c} L_{total}=L_{repr}+w_{1}L_{self}+w_{2}L_{stru}+w_{3}L_{geom} \end{array}$$

In all experiments, we set the loss weights to $w_{1}=0.1$, $w_{2}=1$, and $w_{3}=0.1$ based on empirical tuning. This choice is intended to balance the contributions of each component during training while avoiding overfitting to the pretrained teacher models.

### 3.5. VIO with Depth Estimation

We construct a VIO pipeline based on VINS-RGBD [20], integrating RGB images, the predicted depth maps from the student model, and inertial measurements from an IMU sensor. Given the distinct characteristics of visual and inertial modalities, we apply modality-specific preprocessing steps. The IMU operates at a significantly higher sampling rate than the camera and is subject to considerable sensor noise; thus, we employ pre-integration techniques [19] to fuse the high-rate inertial data effectively. In the visual processing pipeline, feature points are identified in each RGB frame using the Shi–Tomasi corner detection algorithm [25], and their inter-frame correspondences are established via the Kanade–Lucas–Tomasi (KLT) sparse optical flow method [26]. In contrast to conventional RGB-based VIO systems that estimate depth through the perspective-n-point (PnP) algorithm, the proposed method directly incorporates depth values aligned with tracked features, as obtained from the predicted depth map $D_{t}^{s}$. During system initialization, visual–inertial initialization [19] is conducted to jointly estimate the metric scale, gravity direction, and initial pose. Upon successful completion of the initialization phase, subsequent preprocessing outputs are propagated into a sliding-window-based local VIO optimization framework. When a previously visited location is recognized using a Bag-of-Words-based visual retrieval method, the accumulated drift in relative pose estimates is corrected through pose graph optimization. Our experiments demonstrate that the proposed method enhances the robustness of localization in challenging outdoor environments.

## 4. Experiments

### 4.1. Experimental Setup

All experiments were conducted on a workstation equipped with 64 GB RAM, an AMD EPYC 7313P 16-core processor and two NVIDIA RTX 4090 GPUs. The workstation was operated on Ubuntu, with the PyTorch framework [27] and the OpenCV library [28] being primarily utilized. For training Monodepth2 [10], the ResNet-18 encoder [21] pretrained on ImageNet was used to initialize the model parameters. Pretrained weights from the foundation model trained on the meta-dataset [17] were used to initialize both the teacher and student depth networks. The models were optimized using the Adam optimizer [29] with a weight decay of $10^{-2}$, where the initial learning rate was set to $10^{-4}$ for the baseline [10] and $10^{-5}$ for the foundation-based models. To mitigate overfitting, standard data augmentation techniques were employed. These included horizontal flipping and color jittering with brightness, contrast, and saturation factors randomly sampled from the range 0.8 to 1.2 and hue randomly sampled from the range −0.1 to 0.1. Each augmentation was applied with a probability of 50%. Although all sequences were captured at a frame rate of 10 Hz, different temporal triplet configurations were adopted to account for variations in motion dynamics: the triplet [−1, 0, 1] was used for the KITTI dataset, while [−3, 0, 3] was applied to the custom dataset.

Depth estimation was evaluated using three accuracy metrics ($\delta_{1}$, $\delta_{2}$, $\delta_{3}$) and six error metrics (RMSE, RMSEi, AbsRel, SqRel, log10, SIlog). The accuracy metric $\delta_{j}$ denotes the percentage of pixels satisfying $\max\left(\hat{d}/d,d/\hat{d}\right)<1.25^{j}$, where $\hat{d}$ and *d* denote the predicted and ground-truth depth values, respectively. Detailed definitions and formulations of the error metrics are available in previous work [3]. In the tables presenting quantitative results for depth estimation, error metrics and accuracy metrics are indicated with a down arrow and up arrow, respectively. For the odometry evaluation, ground-truth trajectories were generated using a LiDAR-based SLAM algorithm [18], which is known to provide sufficiently reliable estimation performance in outdoor environments rich in features. The evaluation was conducted only on cases where upon returning to the starting position, small objects such as tree poles were mapped at the same location again. As evaluation metrics, we used the relative pose error (RPE) in both translation and rotation and the RMSE of the absolute trajectory error (ATE) as defined in the RGB-D SLAM benchmark [30]. Bold and underlined values indicate the best and second-best performance, respectively, for each task.

### 4.2. KITTI Dataset

To empirically validate the effectiveness of the proposed depth estimation framework, we conduct experiments on the KITTI public dataset [31]. The KITTI dataset was constructed to facilitate a broad spectrum of computer vision tasks, including depth estimation, stereo matching, optical flow, and 3D object detection and tracking. It was collected using a vehicle equipped with stereo cameras, a 3D LiDAR scanner, and GPS/IMU sensors, driving through real-world urban, rural, and highway environments in Karlsruhe, Germany. We employ the Eigen split [3], which is a standardized data partitioning widely adopted for evaluating depth estimation methods. This split comprises 39,810 monocular triplets for training, 4424 for validation, and 697 for evaluation.

**Figure 3:**
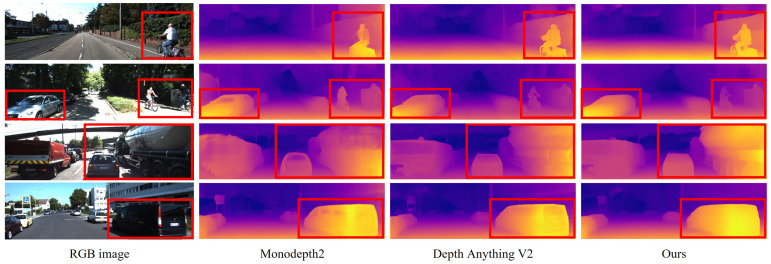
Qualitative comparisons of depth estimation results on the Eigen validation split of the KITTI dataset. The proposed method clearly distinguishes the details of object and recognizes transparent object, as highlighted in the red box.

Quantitative and qualitative evaluations on the KITTI dataset are presented in Table 1 and Figure 3, respectively. We compare the proposed method against Monodepth2 [10] and a fine-tuning strategy that uses pretrained parameters from Depth Anything V2 [9] under a reprojection-based training pipeline. All methods were evaluated using monocular inputs at a fixed resolution of 1024×320 pixels, and median scaling was applied following standard practice. The number of parameters in Depth Anything V2 varies depending on the Vision Transformer [23] backbone: ViT-Large (vitl) yields 335.3 M parameters, while ViT-Small (vits) results in 24.7 M. While the vitl-based model achieves strong performance due to its high representational capacity, its substantially higher computational cost may hinder deployment in resource-constrained settings. In contrast, Monodepth2, with only 14.8 M parameters, is highly efficient in terms of computational resources but requires improvement in estimation accuracy for real-world deployment. The vits-based Depth Anything V2 model achieves better performance than Monodepth2 when fine-tuned, but still falls short of the vitl model overall. Although our proposed method shares the same backbone architecture as the vits-based model, it integrates additional loss functions for training, which lead to the best performance on four error metrics and two accuracy metrics. A slight decrease was observed in AbsRel and $\delta_{1}$, indicating a minor reduction in absolute distance estimation accuracy. However, improvements in SIlog and $\delta_{3}$ suggest that the model has better learned the relative geometric structure within scenes. The qualitative results in Figure 3 further support these findings. In columns 1 and 2, the proposed method better captures fine structures such as pedestrians and bicycles. Furthermore, as shown in columns 3 and 4, the method that relies solely on reprojection loss in the third row exhibits texture-induced artifacts in the predicted depth, whereas the proposed method produces smoother and more geometrically consistent depth maps.

Table 2 presents an ablation analysis that investigates the individual contributions of each loss component in addition to the baseline reprojection loss $L_{repr}$, which is consistently employed across all model variants. To ensure a consistent and equitable evaluation, all experiments were carried out using stereo image inputs with a fixed resolution of 640×192. When incorporating $L_{self}$ or $L_{stru}$ during training, we observed moderate performance gains in SIlog, RMSE, and $\delta_{1}$, indicating improvements in both relative depth accuracy and structural consistency. The adoption of $L_{geom}$, which supervises surface normals, led to performance improvements across four metrics, including AbsRel. The full model trained with all loss terms $L_{total}$ achieved superior performance on most metrics, with only a marginal decline in AbsRel, indicating improved overall depth quality.

### 4.3. Campus Driving Dataset

**Figure 4:**
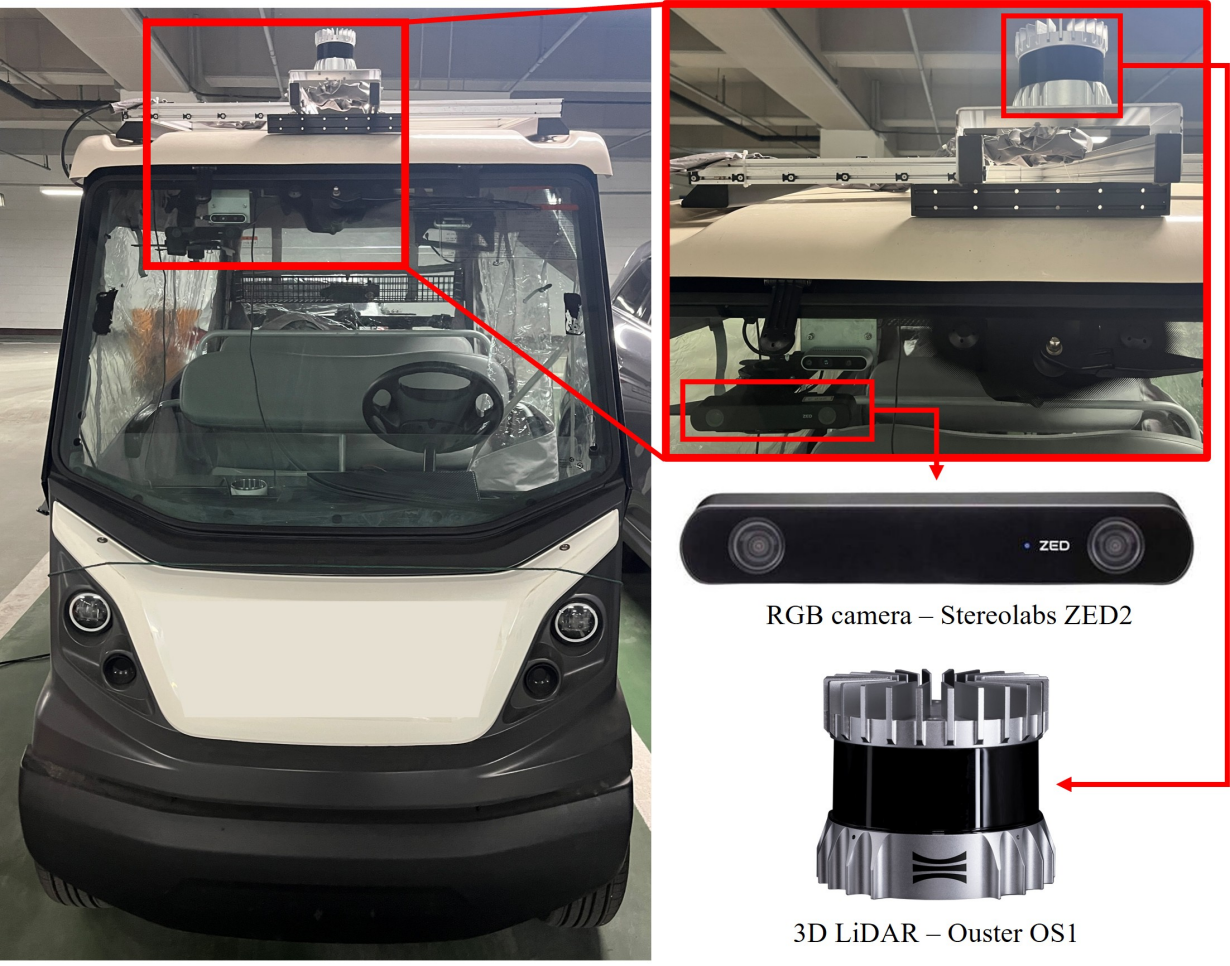
Sensor setup for the custom dataset collection.

We further evaluate the performance of the proposed algorithm on depth and odometry estimation using a custom-collected real-world dataset, and then we compare it against existing methods. As illustrated in Figure 4, the dataset was acquired using a compact electric vehicle equipped with an RGB stereo camera and a 3D LiDAR sensor. The vehicle was driven at speeds ranging from 6 to 12 km/h within a university campus under clear weather conditions. Although the campus is an outdoor environment, it features rich textures and geometric structures in both 3D point clouds and camera images of resolution 640×480, making it suitable for generating reliable ground truth using LiDAR-based SLAM and for validating the application of vision-based SLAM systems. The RGB stereo and LiDAR data were recorded at 10 Hz, while the IMU embedded in the LiDAR system was recorded at 100 Hz. The dataset consists of 12 driving sequences, each lasting between 100 and 400 s. For both tasks, the dataset is partitioned into 8 sequences for training, 1 for validation, and 3 for testing, corresponding to 15,025 stereo pairs for training, 1205 for validation, and 6416 for testing. Reliable ground truth was established through offline extrinsic calibration [32] using optimization-based methods between the camera–IMU and camera–LiDAR sensor pairs.

**Figure 5:**
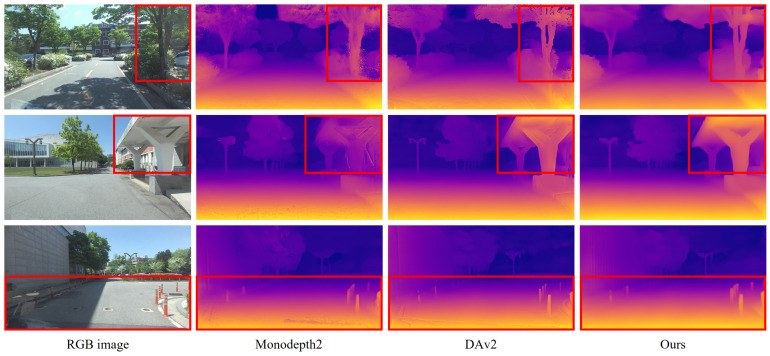
Qualitative comparisons of depth estimation results on the custom dataset. DAv2 denotes Depth Anything V2 [9]. The proposed method clearly distinguishes the details of object, as highlighted in the red box.

Table 3 presents depth estimation results on the custom campus driving dataset, which reveal patterns consistent with those observed on the public dataset. Foundation model-based approaches significantly outperformed Monodepth2 across all evaluation metrics, demonstrating the effectiveness of pretraining on large-scale data. Compared to the baseline fine-tuning strategy using only reprojection loss, the proposed method achieved further improvements, particularly in SIlog and RMSE, indicating enhanced relative and overall depth accuracy. As illustrated in Figure 5, the qualitative comparisons highlight distinct improvements in visual prediction quality. Models trained solely with reprojection loss tend to overfit to image textures, resulting in depth artifacts around regions such as tree foliage and ground shadows. In contrast, the proposed method effectively suppresses such artifacts while providing sharper delineation of structural elements like building pillars and bollard edges along with globally smoother and more coherent depth predictions.

**Figure 6:**
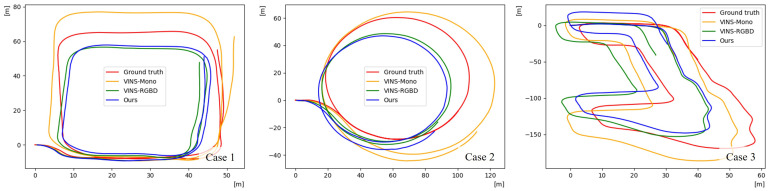
Qualitative comparisons of odometry estimation results on the custom dataset.

As an RGB-based method, the proposed VIO system leverages depth predictions from the student network and is evaluated against both RGB-only [19] and RGB-D [20] VIO baselines, as shown in Table 4. In terms of the RMSE of ATE, which serves as a principal metric for evaluating odometry accuracy, the proposed method consistently outperformed the RGB-only baseline across all test cases, achieving significantly lower error values. In case 1, the proposed method demonstrated slightly superior performance even compared to the RGB-D baseline. Although the translation error of the RPE varied across individual cases, the proposed method achieved a higher average performance. In terms of the rotation error of the RPE, the RGB-based method exhibited slightly better performance than the RGB-D based approach. However, as all VIO methods demonstrated consistently low rotation errors, the differences were not statistically significant. As shown in Figure 6, the trajectory estimated by the existing RGB-based method shows substantial scale errors compared to the ground-truth trajectory. In contrast, the proposed method achieves robust overall odometry estimation, including precise scale estimation, comparable to the performance of the RGB-D-based method in all test cases. These experimental results indicate that the proposed method, despite relying solely on RGB input, could achieve performance comparable to that of the RGB-D approach in outdoor driving environments.

### 4.4. Zero-Shot Depth Estimation

**Figure 7:**
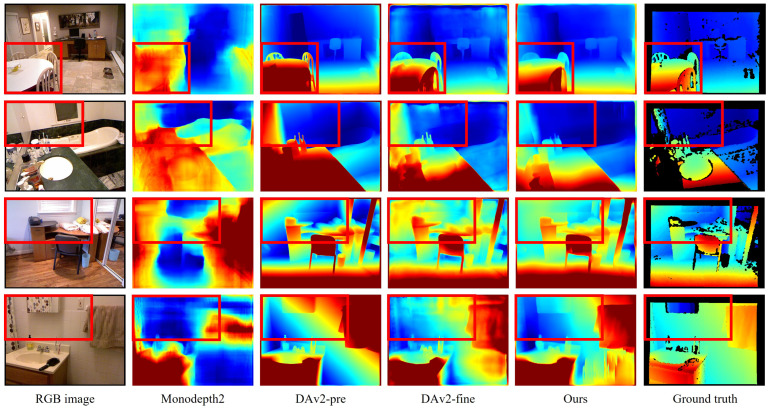
Qualitative comparisons of zero-shot depth estimation results on the NYU dataset. DAv2-pre and DAv2-fine denote the Depth Anything V2 model pretrained by [9] and fine-tuned on KITTI in our work. The proposed method clearly distinguishes the details of object and recognizes transparent object, as highlighted in the red box.

High generalization capability is one of the key characteristics of deep learning models, as it enables faster convergence and higher inference accuracy when fine tuning on a target dataset. To compare the generalization performance of our proposed method with existing approaches, we conducted an ablation study on the NYU dataset [33]. Specifically, we inferred on 654 test images of resolution 640×480 using the Depth Anything V2 model pretrained by [9], as well as monodepth2 [10], Depth Anything V2, and our method, which were all fine-tuned on KITTI. The median scaling was applied to the depth maps predicted by each model, and both qualitative and quantitative evaluations were performed, as shown in Figure 7 and Table 5. With the exception of Monodepth2 [10], the foundation models were observed to produce qualitatively reasonable depth maps. Among them, the pretrained Depth Anything V2 achieved relatively lower performance, partly due to its use of scale-only alignment instead of the original scale-and-shift alignment, as well as the limited handling of transparent objects in the benchmark. Despite the domain difference, the Depth Anything V2 fine-tuned on the real-world KITTI dataset showed substantial quantitative improvements but exhibited qualitatively noisier predictions for low-texture surfaces such as walls and tables. In contrast, the model trained using the proposed method attained the highest scores in six out of nine evaluation metrics and, in qualitative evaluations, generated geometrically consistent predictions that closely matched the ground truth.

## 5. Conclusions

In this study, we fine-tune a pretrained foundation model through a self-supervised learning framework and integrate it into a VIO system, resulting in performance that surpasses existing RGB-based VIO. Our self-supervised training strategy effectively distills knowledge from the pretrained foundation models into a lightweight student depth network, enabling it to inherit the structural understanding learned from large-scale data. In the depth estimation, the proposed method demonstrates notable improvements in qualitative performance. However, metrics related to absolute depth accuracy exhibit slight degradation. In addition, our method requires further experiments to determine optimal loss-function weighting and entails greater complexity in the training pipeline. Nevertheless, the proposed network exhibits robust performance in capturing object boundaries and recognizing transparent surfaces, achieving results comparable to those of the pretrained foundation model. These capabilities cannot be reliably obtained using reprojection-based supervision alone, highlighting the necessity of our knowledge distillation approach. Furthermore, as transparent objects pose inherent challenges not only in depth prediction but also in reliable ground-truth acquisition, future research should investigate both improved estimation techniques and more appropriate evaluation strategies for such regions.

## Figures and Tables

**Table 1:** Quantitative evaluation of depth estimation performance on the Eigen validation split of the KITTI dataset. All methods use monocular input with a resolution of 1024×320. DAv2 denotes Depth Anything V2 [9]. Bold and underline represent the best performance and the next best performance between lightweight models, respectively.

Method	Params	Error Metrics ↓						Accuracy Metrics ↑		
		**SIlog**	**AbsRel**	**SqRel**	**RMSE**	**RMSEi**	**log10**	$\delta_{1}$	$\delta_{2}$	$\delta_{3}$
Monodepth2 [10]	14.8 M	18.293	0.109	0.832	4.648	0.186	0.048	0.888	0.963	0.982
DAv2(vits) [9]	24.7 M	16.994	**0.099 **	0.740	4.314	0.173	**0.043**	**0.908**	**0.969**	0.985
Ours	24.7 M	**16.856**	0.101	**0.687**	**4.280**	**0.172**	0.044	0.899	**0.969**	**0.986**
DAv2(vitl) [9]	335.3 M	16.406	0.090	0.639	4.040	0.166	0.040	0.924	0.971	0.985

**Table 2:** Ablation study of loss function configurations on the Eigen validation split of the KITTI dataset. All methods use stereo input with a resolution of 640×192. Bold and underline represent the best performance and the next best performance, respectively.

$L_{\mathrm{repr}}$	$L_{\mathrm{self}}$	$L_{\mathrm{stru}}$	$L_{\mathrm{geom}}$	SIlog ↓	AbsRel ↓	RMSE ↓	$\delta_{1}$ ↑
✓				19.688	**0.118**	5.230	0.853
✓	✓			18.926	0.120	5.055	0.852
✓		✓		18.872	0.121	5.067	0.854
✓			✓	18.70	0.115	5.024	**0.861**
✓	✓	✓	✓	**18.521**	0.119	**5.017**	**0.861**

**Table 3:** Quantitative evaluation of depth estimation performance on the custom dataset. DAv2 denotes Depth Anything V2 [9]. Bold and underline represent the best performance and the next best performance, respectively.

Method	Error Metrics ↓						Accuracy Metrics ↑		
	**SIlog**	**AbsRel**	**SqRel**	**RMSE**	**RMSEi**	**log10**	$\delta_{1}$	$\delta_{2}$	$\delta_{3}$
Monodepth2 [10]	19.448	0.121	0.810	3.106	0.197	0.049	0.886	0.956	0.979
DAv2(vits) [9]	17.883	**0.096**	0.777	2.997	0.181	**0.039**	**0.918**	**0.964**	0.980
Ours	**17.631**	0.097	**0.712**	**2.857**	**0.180**	0.041	0.903	**0.964**	**0.981**

**Table 4:** Quantitative evaluation of odometry estimation performance on the campus driving dataset. Bold represent the best performance of odometry estimation at the each metric.

Driving Scenario		Case 1	Case 2	Case 3	Average
**Driving Distance [m]**		**318.12**	**394.75**	**502.55**	
**RMSE of ATE [m]**	VINS-Mono [19]	4.9564	8.2617	7.8689	7.0290
	Ours	**4.1027**	**5.2186**	**7.0312**	**5.4508**
	VINS-RGBD [20]	4.3050	4.7788	6.1443	5.0760
**Translation error of RPE [m]**	VINS-Mono [19]	**0.3712**	0.6332	**0.3648**	0.4564
	Ours	0.3993	**0.4627**	0.3921	**0.4180**
	VINS-RGBD [20]	0.3957	0.4092	0.3399	0.3816
**Rotation error of RPE [deg]**	VINS-Mono [19]	**0.3116**	**0.2635**	**0.3291**	**0.3014**
	Ours	0.3319	0.3666	0.3733	0.3573
	VINS-RGBD [20]	0.2975	0.2959	0.3440	0.3125

**Table 5:** Quantitative evaluation of zero-shot depth estimation on the NYU dataset. DAv2-pre and DAv2-fine denote the Depth Anything V2 model pretrained by [9] and fine-tuned on KITTI in our work. Bold and underline represent the best performance and the next best performance, respectively.

Method	Error Metrics ↓						Accuracy Metrics ↑		
	**SIlog**	**AbsRel**	**SqRel**	**RMSE**	**RMSEi**	**log10**	$\delta_{1}$	$\delta_{2}$	$\delta_{3}$
Monodepth2 [10]	35.065	0.336	0.498	1.079	0.370	0.129	0.484	0.778	0.913
DAv2-pre [9]	35.086	0.326	0.787	1.602	0.365	0.124	0.515	0.772	0.981
DAv2-fine [9]	**19.446**	**0.163**	0.160	0.702	**0.203**	**0.068**	0.759	0.958	0.990
Ours	19.464	0.166	**0.140**	**0.661**	**0.203**	0.070	**0.732**	**0.966**	**0.996**

## Data Availability

The KITTI dataset is publicly available online. The public dataset can be found at https://www.cvlibs.net/datasets/kitti, accessed on 10 June 2025.
